# SARS-CoV-2 Infection and COVID-19 Vaccination Associated with Post-Acute Alopecia: Prevalence, Clinical Patterns, and Determinants Among Saudi Adults

**DOI:** 10.3390/v18060613

**Published:** 2026-05-28

**Authors:** Mohammad A. Jareebi, Radwan A. Abutaleb, Norah M. Qassadi, Atheer A. Akoor, Osama A. Mobarki, Shaden S. Alenezi, Jana M. Alsyrwan, Ahlam H. Hakami, Rayim A. Oraybi, Taif Y. Solan, Shatha A. Darbashi, Abdulmohsen J. Almutairi, Saud J. Almutairi, Farjah H. Algahtani, Ghazi I. Al Jowf

**Affiliations:** 1 Family and Community Medicine Department, Faculty of Medicine, Jazan University, Jazan 45142, Saudi Arabia; 2 Department of Dermatology, Jazan General Hospital, Jazan 4514, Saudi Arabia; 3 Faculty of Medicine, Jazan University, Jazan 82817, Saudi Arabia; norahqassadi38@gmail.com (N.M.Q.); osmobarki@gmail.com (O.A.M.); ahlam.hakami055@gmail.com (A.H.H.); rayem990@gmail.com (R.A.O.); taif.solan@gmail.com (T.Y.S.); 4 Ministry of Health, Jazan Health Cluster, Jazan 45142, Saudi Arabia; atheerabdullah01@gmail.com (A.A.A.); shdhy3350@gmail.com (S.A.D.); 5 Faculty of Medicine, Ha’il University, Ha’il 55476, Saudi Arabia; shadensaud136@gmail.com; 6 College of Medicine, King Abdulaziz University, Jeddah 21589, Saudi Arabia; jalsyrawan@stu.kau.edu.sa; 7 Ministry of Health, Qassim Health Cluster, Unaizah 52367, Saudi Arabia; drabdulmohsenalmutairi@gmail.com; 8 College of Medicine, Shaqra University, Riyadh 11961, Saudi Arabia; saudjasser1@gmail.com; 9 Department of Medicine, Oncology Center, Chair of Epidemiology and Public Health Research, Faculty of Medicine, King Saud University/King Saud Medical City, Riyadh 12373, Saudi Arabia; falgahtani@ksu.edu.sa; 10 Department of Public Health, College of Applied Medical Sciences, University Medical Clinics Complex, King Faisal University, Al Hofuf 37912, Saudi Arabia

**Keywords:** SARS-CoV-2, COVID-19 complications, alopecia, telogen effluvium, COVID-19 vaccination, immune-mediated hair loss, nutritional deficiency

## Abstract

**Background/Objectives**: SARS-CoV-2 infection and COVID-19 vaccination have both been linked to post-acute alopecia, yet prevalence, patterns, and correlates remain poorly defined. We aimed to determine the prevalence and patterns of alopecia after SARS-CoV-2 infection and COVID-19 vaccination, and to identify independent correlates among Saudi adults. **Methods**: A cross-sectional study of 1261 Saudi adults (≥18 years) was conducted nationwide between March and July 2025 using a structured, self-administered, bilingual (Arabic/English) online questionnaire distributed via social media. Eligible participants had received at least one COVID-19 vaccine dose. Data captured demographics, vaccination type and dose history, prior SARS-CoV-2 infection, self-reported alopecia subtype guided by plain-language definitions, comorbidities, and nutritional status. Hair loss and subtypes were entirely self-reported and not clinically or dermoscopically confirmed. Chi-square tests assessed univariate associations, and multivariable logistic regression identified independent predictors of any self-reported hair loss. **Results**: Mean age was 28.0 ± 9.6 years; 62.1% were female, 97.7% vaccinated (76.0% Pfizer-BioNTech first dose, 58.1% three doses), and 53.6% reported prior COVID-19 infection. Overall self-reported hair loss prevalence was 62.4%, with hair loss reported by 57.5% of vaccinated and 49.7% of COVID-positive participants. Self-reported telogen effluvium was the predominant pattern (56.3%), followed by androgenetic alopecia (39.3%) and alopecia areata (2.7%). Independent correlates were female sex (aOR 4.89; 95% CI 3.78–6.32), vitamin/mineral deficiency (aOR 2.31; 1.73–3.09), iron deficiency anemia (aOR 1.89; 1.29–2.78), prior SARS-CoV-2 infection (aOR 1.31; 1.02–1.68), and COVID-19 vaccination (aOR 7.45; 2.51–22.12). **Conclusions**: Self-reported hair loss was reported by nearly two-thirds of Saudi adults after COVID-19 vaccination or infection. Female sex and correctable nutritional deficiencies are the strongest modifiable correlates and warrant targeted screening and counseling.

## 1. Introduction

Saudi Arabia has deployed multiple COVID-19 vaccines, including mRNA-based (Pfizer/BioNTech and Moderna) and viral vector (Oxford/AstraZeneca and Johnson & Johnson) platforms, as part of its mass vaccination campaign, with roughly 70% of the Saudi population having received at least one dose by early 2023 [1]. Hair loss disorders are broadly categorized by mechanisms. Alopecia areata (AA) is an autoimmune non-scarring hair loss characterized by well-circumscribed patches of complete hair loss on the scalp or body [2]. Telogen effluvium (TE) is a diffuse, non-scarring shedding that typically occurs months after a triggering event such as severe illness, fever, or psychological stress, and usually improves within 6–12 months unless the trigger persists.

Hair loss reported after COVID-19 vaccination has been increasingly documented, though its true incidence remains poorly established. Pharmacovigilance systems have captured only modest numbers: the UK MHRA logged 154 reports of AA after COVID-19 vaccines, and the U.S. VAERS system logged 126 such reports by late 2022, representing a tiny fraction of doses administered [3]. Cross-sectional surveys report higher self-reported rates: a Saudi survey of 726 vaccinated adults found that 67.6% noticed some shedding after vaccination, although only 27.6% attributed it to the vaccine [3]. An Egyptian dermoscopic study identified clinical TE in 23.9% of vaccine recipients within 2–3 months after vaccination [4]. Reported rates across populations range widely (approximately 24–68%), reflecting methodological heterogeneity and the difficulty of establishing causality [4,5].

Hair loss can be psychologically burdensome and significantly impair wellbeing. Patients consistently experience anxiety, depression, and social distress disproportionate to the objective amount of hair loss [6]. Women with severe AA report markedly worse quality-of-life scores than those with mild disease [7], and a recent study in the Eastern Province of Saudi Arabia found that more than 50% of patients with alopecia had high depression scores and reduced quality of life [8]. Any vaccine- or infection-related hair loss therefore carries meaningful psychosocial consequences affecting self-image and daily functioning [6,8].

Emerging literature describes sporadic cases of hair loss after COVID-19 vaccination. Hernández-Arroyo et al. reported five cases of TE or alopecia universalis occurring days to weeks after mixed mRNA/viral-vector regimens [9]. A systematic review identified only 51 patients with new-onset or relapsed AA after COVID-19 vaccines worldwide [10]. Of 154 UK Yellow Card reports of post-vaccine alopecia, 50% followed Pfizer/BioNTech and 40% followed AstraZeneca [5]. Collectively, peer-reviewed evidence suggests a possible association between COVID-19 vaccination and new or worsened alopecia in susceptible individuals, but such events appear uncommon and most reports derive from small studies or passive surveillance [5,10].

Despite these data, significant gaps persist in the Saudi context. No published population-based studies quantify the prevalence of AA or TE after COVID-19 vaccination, nor is there information on how vaccine type or dose may influence risk. Existing Saudi data are limited to a single cross-sectional survey of TE symptoms [3]. Clinicians and public health officials therefore lack evidence on the burden of vaccine- or infection-associated alopecia in Saudi Arabia and on whether specific demographic or comorbidity subgroups are more vulnerable.

The present study addresses these gaps through a cross-sectional analysis of Saudi adults. We estimated the prevalence of self-reported hair loss (distinguishing alopecia areata from telogen effluvium) following COVID-19 immunization and infection, and identified correlates with vaccine type, dosage, and patient characteristics. Given the cross-sectional design, all associations reported reflect perceived temporal relationships between exposures and self-reported outcomes and should not be interpreted as evidence of causation.

## 2. Materials and Methods

### 2.1. Study Design and Setting

A cross-sectional design was adopted to estimate the prevalence of hair loss and identify its associations within a defined time frame, in line with established methodological guidance for prevalence studies [11]. The study explored the prevalence of hair loss following COVID-19 vaccination and assessed its predictors among adults in Saudi Arabia.

### 2.2. Study Population and Sampling

We included Saudi residents aged ≥18 years who had received at least one dose of a COVID-19 vaccine (Pfizer-BioNTech, Moderna, AstraZeneca, or Johnson & Johnson). Convenience sampling was used, and participants provided informed consent without further restrictions. Recruitment via social media platforms likely enriched the sample for younger, more digitally engaged, and more highly educated individuals, and potentially for those already concerned about or experiencing hair loss, given the explicitly hair-loss-focused framing of the survey. These factors introduce a substantial risk of self-selection bias that may inflate observed prevalence estimates relative to the broader Saudi adult population.

The minimum required sample size was estimated at 385 participants, based on a 95% confidence level, 5% margin of error, and 50% expected prevalence, using the Raosoft sample size calculator and Saudi census data. This minimum was increased to 424 after adding 10% for anticipated missing data. To improve statistical precision, a target of 1000 participants was set. The study ultimately received 1262 responses, of which 1261 met eligibility and were included in the final analysis, exceeding the minimum requirement. Generalizability to the broader Saudi adult population remains limited by the non-representative demographic composition of the convenience sample. All individuals aged ≥18 years who had received at least one COVID-19 vaccine dose were eligible, regardless of gender, socioeconomic status, or educational level. Those who refused to participate or were unable to provide informed consent were excluded.

### 2.3. Data Collection Tools

#### 2.3.1. Questionnaire Structure

A structured, self-administered questionnaire was developed in Google Forms and distributed in Arabic and English. The instrument comprised 82 items across four domains: demographics, COVID-19 vaccination history, hair loss assessment, and free-text comments. Demographic items captured age, gender, income, education, residence, medical and family history. Vaccination items recorded vaccine type, number of doses, and time since last dose. Hair loss items recorded onset, pattern, severity, perceived trigger, and any pre-vaccination history. A pilot study with 20 participants confirmed clarity and reliability before full distribution.

#### 2.3.2. Classification of Alopecia Subtypes

Alopecia subtypes were classified from participant responses to plain-language descriptions developed for non-specialist audiences. Telogen effluvium was described as diffuse generalized shedding across the scalp, androgenetic alopecia as gradual patterned thinning at the crown or hairline, and alopecia areata as discrete patchy areas of complete hair loss. Participants selected the description most closely matching their experience. Validated dermatological tools such as the Sinclair Scale, the Severity of Alopecia Tool (SALT), and dermoscopic criteria were not applied, given the online format and the absence of in-person examination; the resulting subtype distribution should therefore be interpreted as participant-perceived patterns rather than clinically confirmed diagnoses.

#### 2.3.3. Outcome Definition

The primary outcome was any self-reported hair loss, regardless of attributed cause or temporal relationship to vaccination or infection. These composite captured cases attributed to vaccination, prior COVID-19 infection, stress, dietary factors, and those without an identified trigger; these subcategories were not mutually exclusive.

#### 2.3.4. Temporal Definitions

Post-vaccine hair loss was defined as new or worsening shedding within twelve months of the most recent vaccine dose, with onset categorized as within one month, one to three months, three to six months, or beyond six months. The same twelve-month window was applied to post-infection cases. As telogen effluvium typically develops 6 to 16 weeks after a trigger, this window was chosen to capture the full clinically plausible interval.

### 2.4. Data Collection Process

Data collection was conducted entirely online from March to July 2025 through WhatsApp, Telegram, and similar platforms. Informed consent was obtained electronically before enrolment, and participation was voluntary and anonymous. Duplicate or incomplete submissions were identified and resolved by routine review. A total of 1262 valid responses were received and 1261 met eligibility criteria.

### 2.5. Data Analysis

Analyses were performed in RStudio (R 4.2.3, R Foundation for Statistical Computing, Vienna, Austria). The primary outcome in all logistic regression models was defined as any self-reported hair loss versus no hair loss. COVID-19 vaccination status and prior infection status were entered as independent exposure variables rather than definitional components of the outcome. Stress-related, diet-related, and trigger-unidentified cases were all captured under this binary outcome.

Categorical data are presented as frequencies and percentages, and continuous variables as means ± SD. Normality was assessed by Shapiro–Wilk tests. Chi-square tests examined categorical associations, and t-tests, or ANOVA, were used for continuous outcomes. Multivariable logistic regression identified independent predictors of hair loss, adjusting for confounders; results are expressed as odds ratios (OR) with 95% confidence intervals (CI). Statistical significance was set at *p* < 0.05. The dataset contained no missing values for the primary outcome, exposures, or covariates, so complete-case analysis was used. Multicollinearity was assessed by variance inflation factors (VIFs), with values < 5 considered acceptable, and overall fit was evaluated using McFadden’s pseudo-R^2^.

### 2.6. Ethical Approval

The study received approval from the Local Committee for Research Ethics at Jazan University (reference No.: REC-46/06/1243, dated 24 December 2024). Informed consent was obtained electronically from all participants. Data confidentiality was maintained through password-protected databases accessible only to the research team.

### 2.7. Use of Generative Artificial Intelligence

Generative AI was used solely for language editing. Study design, data, analysis, and interpretation were entirely conducted by the authors.

## 3. Results

### 3.1. Baseline Characteristics

A total of 1261 participants were included. Mean age was 28.0 ± 9.6 years, and 62.1% (n = 783) were female. Most were Saudi nationals (91.4%), urban residents (74.7%), and held a bachelor’s degree (73.6%). Students comprised 51.8% of the sample, and 58.5% were from the Southern region. Reported comorbidities included vitamin/mineral deficiency (34.3%), iron deficiency anemia (17.8%), diabetes mellitus (6.9%), hypertension (5.3%), and hypothyroidism (4.4%). Physical activity was reported by 44.0%, and 16.3% were current smokers (Table 1).

### 3.2. COVID-19 Exposure and Vaccination

A history of COVID-19 infection was reported by 53.6% (n = 676) of participants, and 97.7% (n = 1232) were vaccinated. Among vaccinated participants, 58.1% had received three doses, 33.7% two doses, and 8.2% a single dose. Pfizer-BioNTech was the most common first-dose vaccine (76.0%), followed by Moderna (15.3%), AstraZeneca (7.7%), and Johnson & Johnson (1.0%). A single vaccine type was used in 63.9% of vaccinated participants, while 36.1% received a combination (Table 2).

### 3.3. Prevalence and Patterns of Self-Reported Hair Loss

Self-reported hair loss was reported by 62.4% (n = 787) of participants. Among COVID-positive participants, 49.7% (n = 336) reported post-infection hair loss; among vaccinated participants, 57.5% (n = 709) reported post-vaccination hair loss. Stress-related hair loss was reported by 34.4% (n = 434) and diet-related hair loss by 15.3% (n = 193); 50.3% (n = 634) reported no identifiable trigger. These categories were not mutually exclusive.

Among participants reporting hair loss (n = 787), the predominant self-reported pattern was telogen effluvium (56.3%, n = 443), followed by androgenetic alopecia (39.3%, n = 309) and alopecia areata (2.7%, n = 21); 1.8% (n = 14) reported other or unspecified patterns (Table 3).

### 3.4. Onset and Timing of Hair Loss

**Figure 1:**
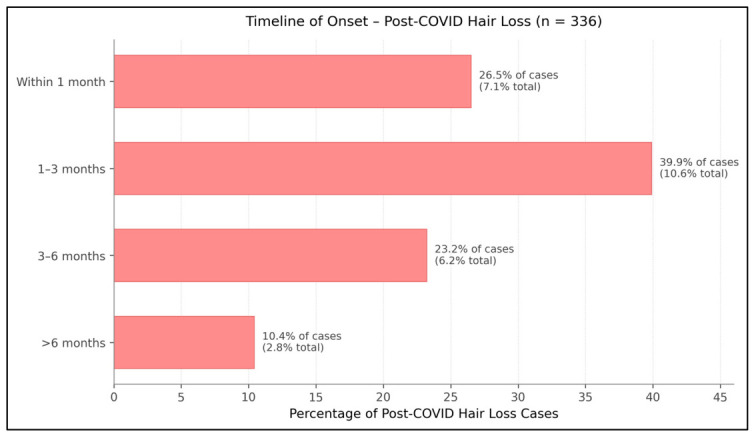
Temporal distribution of hair loss onset following COVID-19 infection (n = 336). The x-axis represents time intervals from confirmed or suspected COVID-19 infection to self-reported hair loss onset (within 1 month, 1–3 months, 3–6 months, and beyond 6 months); the y-axis represents number of participants.

**Figure 2:**
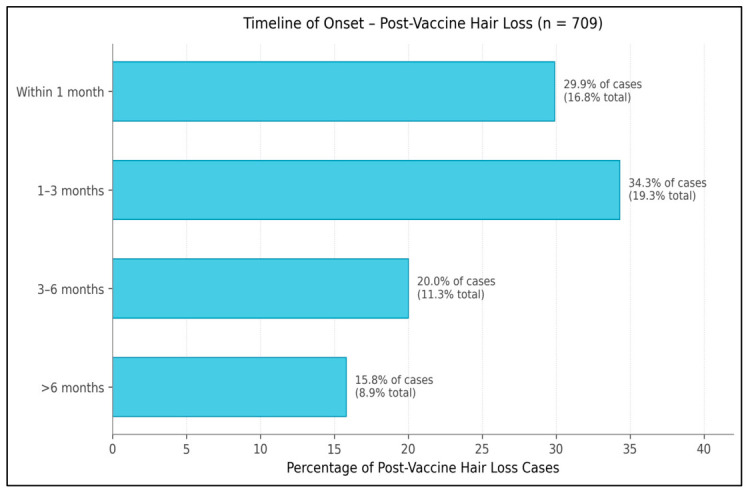
Temporal distribution of hair loss reported after COVID-19 vaccination (n = 709). The x-axis represents time intervals from the most recent vaccine dose to self-reported hair loss onset (within 1 month, 1–3 months, 3–6 months, and beyond 6 months); the y-axis represents number of participants.

Among 336 participants reporting post-infection hair loss, onset occurred most often between one and three months after infection. Among 709 participants reporting post-vaccination hair loss, the largest proportion reported onset within the first month after vaccination, with smaller proportions at later intervals (Figure 1 and Figure 2).

### 3.5. Factors Associated with Hair Loss

In univariate analysis, hair loss was significantly associated with female sex (OR 5.63; 95% CI 4.50–7.04; *p* < 0.001), prior COVID-19 infection (OR 1.73; 1.39–2.15; *p* < 0.001), COVID-19 vaccination (OR 10.89; 3.78–31.42; *p* < 0.001), bachelor’s-level education (OR 2.52; 1.94–3.27; *p* < 0.001), diabetes (OR 1.73; 1.06–2.84; *p* = 0.029), iron deficiency anemia (OR 2.50; 1.77–3.52; *p* < 0.001), vitamin/mineral deficiency (OR 3.00; 2.31–3.90; *p* < 0.001), hypothyroidism (OR 2.49; 1.27–4.89; *p* = 0.007), and current smoking (OR 1.39; 1.01–1.91; *p* = 0.044). Physical activity was inversely associated with hair loss (OR 0.75; 0.59–0.94; *p* = 0.012). Full univariate results are shown in Table 4.

**Figure 3:**
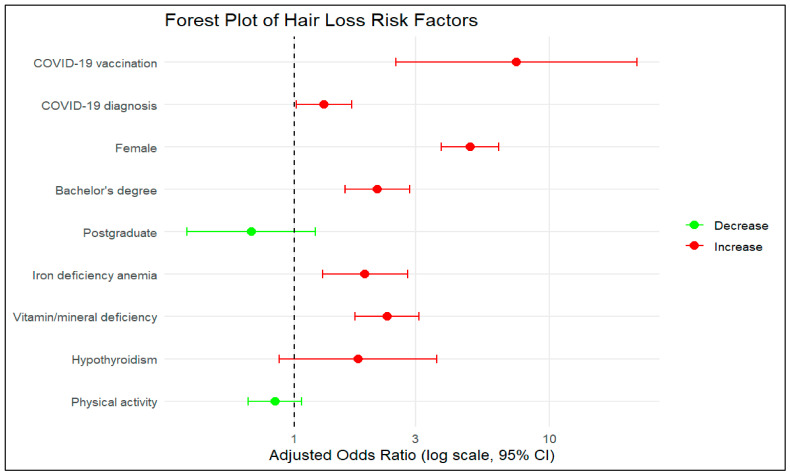
Forest plot of adjusted odds ratios for factors independently associated with self-reported hair loss in multivariable logistic regression. Points represent adjusted odds ratios, and horizontal lines represent 95% confidence intervals. The vertical reference line indicates aOR = 1; confidence intervals that do not cross this line are statistically significant at *p* < 0.05. The x-axis is shown on a logarithmic scale.

In multivariable logistic regression, independent factors associated with hair loss were female sex (aOR 4.89; 3.78–6.32; *p* < 0.001), COVID-19 vaccination (aOR 7.45; 2.51–22.12; *p* < 0.001), prior COVID-19 infection (aOR 1.31; 1.02–1.68; *p* = 0.033), vitamin/mineral deficiency (aOR 2.31; 1.73–3.09; *p* < 0.001), iron deficiency anemia (aOR 1.89; 1.29–2.78; *p* = 0.001), and bachelor’s-level education (aOR 2.12; 1.58–2.84; *p* < 0.001). All variance inflation factors were below 1.25, and McFadden’s pseudo-R^2^ was 0.176 (Table 5; Figure 3).

## 4. Discussion

### 4.1. Key Findings

In this cross-sectional study of 1261 Saudi adults, this work provides evidence on the perceived burden of hair loss following COVID-19 vaccination and SARS-CoV-2 infection, with self-reported hair loss affecting 62.4% of the cohort and telogen effluvium the predominant pattern (56.3% of those with hair loss). Multivariable analysis identified female sex, COVID-19 vaccination, prior SARS-CoV-2 infection, vitamin/mineral deficiency, iron deficiency anemia, and bachelor’s-level education as independent factors associated with hair loss. To our knowledge, this is one of the few studies in the region to evaluate vaccination and infection within a single analytical framework, identifying modifiable nutritional correlates directly relevant to dermatology and primary care practice.

### 4.2. Prevalence in Context

The 57.5% prevalence of post-vaccination hair loss in our cohort is broadly consistent with an earlier Saudi survey (67.6%) [3], and the 49.7% post-infection rate aligns with another Saudi report (59.7%) [12]. Self-reported survey prevalence consistently exceeds estimates from pharmacovigilance and dermoscopy-based studies [4,13,14], reflecting differences in case definition and ascertainment between population-level surveys and clinical or passive surveillance systems. The 62.4% overall figure should therefore be read as the upper end of the reported range and as a measure of perceived burden in an online cohort, rather than a clinically confirmed incidence. That distinction itself is meaningful: perceived burden drives healthcare-seeking behavior, patient anxiety, and clinical workload, and quantifying it has independent public-health value alongside dermoscopy-based incidence estimates.

### 4.3. Comparative Risk: Vaccination, Infection, and the Broader Evidence

A nationwide Korean study of more than 5.7 million individuals reported a positive association between COVID-19 vaccination and hair loss disorders (TE aOR 1.495; AA aOR 1.243), with no significant residual association for infection after adjustment [15]. Our directionally consistent findings are compatible with a transient vaccine-associated inflammatory pathway warranting prospective confirmation [16]. The magnitude of our vaccination estimate (aOR 7.45) is best interpreted alongside this body of work, since the unvaccinated comparator in our sample (n = 29) limits precision even though the direction of effect is concordant with the much larger Korean cohort. Future studies with larger unvaccinated groups, ideally using Firth’s penalized likelihood or exact logistic regression, would refine the magnitude.

The differential attribution of hair loss to vaccination rather than infection in self-report studies is itself informative. Pervasive online discourse linking COVID-19 vaccines to adverse outcomes [5] may shape causal attributions in surveys of this kind, underscoring the importance of pairing survey data with pharmacovigilance and dermoscopically confirmed clinical evidence before drawing public-health conclusions. Quantifying this perception–evidence gap is a useful contribution of survey-based work and provides a baseline against which future objective measurements can be benchmarked.

### 4.4. Predominant Pattern and Proposed Mechanisms

The predominance of telogen effluvium in our cohort is consistent with the recognized pathophysiology of stress-induced shedding, in which a synchronized, premature transition of follicles from anagen to telogen follows a systemic trigger by several weeks to months [3,17]. For post-vaccination cases, a plausible mechanism involves a transient surge of pro-inflammatory cytokines such as IFN-γ and IL-6, which can disrupt follicular cycling and shorten the anagen phase [4,17]. For post-infection cases, contributing factors include direct viral effects, fever, physiological stress, and systemic inflammation, all well-described features of acute and post-acute COVID-19 [12,17]. The smaller alopecia areata fraction (2.7%) is consistent with autoimmune disruption of follicular immune privilege in genetically predisposed individuals, in whom systemic immune activation may precipitate disease onset or relapse [4,14,16]. Confirmation will require histopathological and immunological studies linking serum cytokine profiles, scalp biopsy findings, and trichoscopic measures to exposure history.

### 4.5. Comorbidities and Lifestyle

Several comorbidities and lifestyle factors showed meaningful associations. Hypothyroidism was associated with increased univariate odds (OR 2.49; *p* = 0.007), consistent with the established link between thyroid dysfunction and telogen effluvium, although it did not retain independent significance after adjustment (aOR 1.78). Diabetes mellitus (OR 1.73; *p* = 0.029) may reflect metabolic and microvascular effects on the follicular cycle, particularly in poorly controlled diseases. Physical activity was inversely associated in univariate analysis (OR 0.75; *p* = 0.012), and current smoking was associated with marginally increased odds (OR 1.39; *p* = 0.044), in line with the literature on oxidative stress and microvascular contributions to follicular miniaturization. These findings indicate that the hair loss burden in this population is shaped not only by COVID-19 exposures but also by underlying metabolic and lifestyle factors amenable to clinical intervention and that should be considered during workup.

### 4.6. Modifiable Determinants: Female Sex and Nutritional Status

Female sex was the strongest demographic correlate (aOR 4.89), in keeping with the wider literature on post-COVID and classic telogen effluvium, where women of reproductive age are disproportionately affected by hormonal, metabolic, and psychosocial factors that influence the hair cycle [3,12]. The most clinically actionable signal, however, lies in the robust independent associations with iron deficiency anemia (aOR 1.89) and vitamin/mineral deficiency (aOR 2.31). These conditions are common in the regional population, easily screened for using a basic laboratory panel, and directly correctable through dietary modification and supplementation. They are therefore a natural target for routine assessment in patients presenting with post-COVID or post-vaccination hair loss. Optimizing nutritional status is a low-cost intervention that may meaningfully shorten the course of telogen effluvium regardless of trigger and represents the most immediately translatable finding of this study.

### 4.7. Clinical Implications

Hair loss carries a psychosocial burden disproportionate to its objective severity [6,8]. Primary care physicians and dermatologists should therefore take post-COVID and post-vaccination hair loss complaints seriously, with three practical priorities. First, targeted screening for iron status, vitamin D, and other micronutrients should form part of the initial assessment, given the strong nutritional signal in this and other cohorts. Second, dermatological evaluation, including trichoscopy where available, supports accurate clinical confirmation of subtype and informs prognosis. Third, reassurance about the typically self-limiting course of telogen effluvium, where most cases resolve within six months once the trigger is removed, is a key element of management [4,12]. This combination of investigation, accurate diagnosis, and informed counselling supports patients while reducing inappropriate causal attribution to vaccination and easing the clinical workload generated by unmanaged anxiety.

### 4.8. Strengths and Limitations

The principal strengths of this study are the large sample (n = 1261), the simultaneous evaluation of vaccine- and infection-associated hair loss within a single cohort, the use of multivariable adjustment with formal model diagnostics (all VIFs below 1.25; McFadden’s pseudo-R^2^ = 0.176), and the provision of regional epidemiological data from a setting with limited prior evidence.

Several limitations should be acknowledged, alongside the steps taken to mitigate them. The cross-sectional design cannot establish causality; all associations are therefore framed as correlates, and temporal descriptors refer only to participant recall. Online recruitment introduces self-selection bias; we mitigated this with bilingual distribution, a sample well above the calculated minimum (n = 1261 versus 424 required), and transparent reporting of the cohort’s demographic profile. The unvaccinated reference group was small (n = 29); the vaccination estimate is therefore interpreted alongside the much larger Korean cohort [15] rather than in isolation. Hair loss subtypes were classified from plain-language descriptions rather than trichoscopy; a piloted instrument with non-overlapping definitions was used to minimize misclassification, and subtypes are presented as participant-perceived patterns. Recall and attribution bias are inherent to retrospective self-report; this was addressed in part by analyzing any self-reported hair loss as the primary outcome, independent of participant-attributed cause. Finally, some confounders were not captured (hormonal contraceptive use, pregnancy or postpartum status, psychiatric history, concurrent medications); these are flagged as priorities for future prospective work with dermatologist-confirmed diagnoses and pre-exposure baseline assessment.

## 5. Conclusions

In this nationwide cross-sectional study of Saudi adults, self-reported hair loss was a common experience after COVID-19 vaccination and infection, with telogen effluvium the predominant pattern. Female sex, iron deficiency anemia, and vitamin/mineral deficiency were the strongest independent correlates and define a clear, low-cost clinical workup for affected patients: targeted nutritional screening, dermatological confirmation of subtype, and reassurance about the typically self-limiting course of telogen effluvium. Prospective, dermatologist-confirmed studies with adequate unexposed comparators are now needed to refine incidence estimates and establish causal direction.

## Figures and Tables

**Table 1:** Baseline Characteristics of Study Participants (N = 1261).

Characteristic	n (%)
Demographics	
Gender	
*Male*	478 (37.9%)
*Female*	783 (62.1%)
Age, mean ± SD (years)	28.0 ± 9.6
Nationality	
*Saudi*	1153 (91.4%)
*Non-Saudi*	108 (8.6%)
Residence	
*Urban*	942 (74.7%)
*Rural*	319 (25.3%)
Education	
*High school or less*	261 (20.7%)
*Bachelor’s degree*	928 (73.6%)
*Postgraduate*	72 (5.7%)
Occupation	
*Student*	653 (51.8%)
*Employed*	516 (40.9%)
*Unemployed*	92 (7.3%)
Region	
*Southern*	738 (58.5%)
*Central*	284 (22.5%)
*Western*	151 (12.0%)
*Eastern*	88 (7.0%)
**Health Status**	
*Iron deficiency anemia*	225 (17.8%)
*Vitamin/mineral deficiency*	433 (34.3%)
*Diabetes mellitus*	87 (6.9%)
*Hypertension*	67 (5.3%)
*Hypothyroidism*	55 (4.4%)
**Lifestyle Factors**	
*Physical activity*	555 (44.0%)
*Current smoking*	206 (16.3%)
*Ex-smoking*	74 (5.9%)

**Table 2:** COVID-19 Exposure and Vaccination Characteristics (N = 1261).

Characteristic	n (%)
COVID-19 Diagnosis	
*Yes*	676 (53.6%)
*No*	585 (46.4%)
**COVID-19 Vaccination Status**	
*Vaccinated*	1232 (97.7%)
*Unvaccinated*	29 (2.3%)
**Vaccination Schedule (among vaccinated, n = 1232)**	
*One dose*	101 (8.2%)
*Two doses*	415 (33.7%)
*Three doses*	716 (58.1%)
**First Dose Vaccine Type (n = 1232)**	
*Pfizer-BioNTech*	936 (76.0%)
*Moderna*	189 (15.3%)
*AstraZeneca*	95 (7.7%)
*Johnson & Johnson*	12 (1.0%)
**Vaccine Type Pattern (n = 1232)**	
*Single vaccine type*	787 (63.9%)
*Combination of vaccines*	445 (36.1%)

**Table 3:** Hair Loss Status and Characteristics (N = 1261).

Hair Loss Category	n (%)
Overall Hair Loss Status	
*Hair loss*	787 (62.4%)
*No hair loss*	474 (37.6%)
**Hair Loss by Exposure Status**	
**Hair loss reported after COVID-19 infection:**	
*Among COVID-positive participants (n = 676)*	336 (49.7%)
*Among COVID-negative participants (n = 585)*	0 (0%)
**Hair loss reported after vaccination:**	
*Among vaccinated participants (n = 1232)*	709 (57.5%)
*Among unvaccinated participants (n = 29)*	0 (0%)
**Hair Loss Patterns (among those with hair loss, n = 787)**	
*Telogen effluvium*	443 (56.3% of cases; 35.1% of total)
*Androgenetic alopecia*	309 (39.3% of cases; 24.5% of total)
*Alopecia areata*	21 (2.7% of cases; 1.7% of total)
*Other/unspecified*	14 (1.8% of cases; 1.1% of total)
**Associated Factors**	
*Stress-related hair loss*	434 (34.4% of total sample)
*Diet-related hair loss*	193 (15.3% of total sample)
*No identifiable trigger*	634 (50.3% of total sample)

**Table 4:** Univariate Analysis of Risk Factors for Any Hair Loss (N = 1261).

Variable	Category	No Hair Loss	Hair Loss	OR (95% CI)	*p*-Value
		n = 474 (%)	n = 787 (%)		
Demographics					
Gender	*Male*	297 (62.1%)	181 (37.9%)	1.00 (ref)	-
	*Female*	177 (22.6%)	606 (77.4%)	5.63 (4.50–7.04)	<0.001
Age (years)	*Mean ± SD*	27.0 ± 9.2	28.3 ± 9.8	1.01 (1.00–1.03)	0.015
Nationality	*Saudi*	428 (37.1%)	725 (62.9%)	1.00 (ref)	-
	*Non-Saudi*	46 (42.6%)	62 (57.4%)	0.80 (0.53–1.20)	0.279
Education	*High school or less*	139 (53.3%)	122 (46.7%)	1.00 (ref)	-
	*Bachelor’s degree*	289 (31.1%)	639 (68.9%)	2.52 (1.94–3.27)	<0.001
	*Postgraduate*	46 (63.9%)	26 (36.1%)	0.64 (0.37–1.12)	0.119
**COVID-19 Related Exposures**					
COVID-19 diagnosis	*No*	260 (44.4%)	325 (55.6%)	1.00 (ref)	-
	*Yes*	214 (31.7%)	462 (68.3%)	1.73 (1.39–2.15)	<0.001
COVID-19 vaccination	*No*	25 (86.2%)	4 (13.8%)	1.00 (ref)	-
	*Yes*	449 (36.4%)	783 (63.6%)	10.89 (3.78–31.42)	<0.001
**Clinical Conditions**					
Diabetes mellitus	*No*	451 (38.4%)	723 (61.6%)	1.00 (ref)	-
	*Yes*	23 (26.4%)	64 (73.6%)	1.73 (1.06–2.84)	0.029
Hypertension	*No*	456 (38.2%)	738 (61.8%)	1.00 (ref)	-
	*Yes*	18 (26.9%)	49 (73.1%)	1.68 (0.96–2.95)	0.068
Iron deficiency anemia	*No*	425 (41.0%)	611 (59.0%)	1.00 (ref)	-
	*Yes*	49 (21.8%)	176 (78.2%)	2.50 (1.77–3.52)	<0.001
Vitamin/mineral deficiency	*No*	379 (45.8%)	449 (54.2%)	1.00 (ref)	-
	*Yes*	95 (21.9%)	338 (78.1%)	3.00 (2.31–3.90)	<0.001
Hypothyroidism	*No*	463 (38.4%)	743 (61.6%)	1.00 (ref)	-
	*Yes*	11 (20.0%)	44 (80.0%)	2.49 (1.27–4.89)	0.007
**Lifestyle Factors**					
Physical activity	*No*	244 (34.6%)	462 (65.4%)	1.00 (ref)	-
	*Yes*	230 (41.4%)	325 (58.6%)	0.75 (0.59–0.94)	0.012
Smoking status	*Non-smoker*	378 (38.5%)	603 (61.5%)	1.00 (ref)	-
	*Ex-smoker*	32 (43.2%)	42 (56.8%)	0.82 (0.51–1.33)	0.426
	*Current smoker*	64 (31.1%)	142 (68.9%)	1.39 (1.01–1.91)	0.044

**Table 5:** Multivariable Logistic Regression Analysis for Any Hair Loss Risk Factors.

Variable	Category	Adjusted OR	95% CI	*p*-Value
COVID-19 Related Factors				
COVID-19 diagnosis	*No*	1.00 (ref)	-	-
	*Yes*	1.31	1.02–1.68	0.033
COVID-19 vaccination *	*No*	1.00 (ref)	-	-
	*Yes*	7.45	2.51–22.12	<0.001
**Demographics**				
Gender	*Male*	1.00 (ref)	-	-
	*Female*	4.89	3.78–6.32	<0.001
Age (years)	*-*	1.01	0.99–1.03	0.142
Education	*High school or less*	1.00 (ref)	-	-
	*Bachelor’s degree*	2.12	1.58–2.84	<0.001
	*Postgraduate*	0.68	0.38–1.21	0.189
**Clinical Conditions**				
Iron deficiency anemia	*No*	1.00 (ref)	-	-
	*Yes*	1.89	1.29–2.78	0.001
Vitamin/mineral deficiency	*No*	1.00 (ref)	-	-
	*Yes*	2.31	1.73–3.09	<0.001
Hypothyroidism	*No*	1.00 (ref)	-	-
	*Yes*	1.78	0.87–3.63	0.114
**Lifestyle Factors**				
Physical activity	*No*	1.00 (ref)	-	-
	*Yes*	0.84	0.66–1.07	0.158

* The vaccination aOR is driven by a very small unvaccinated reference group (n = 29) with only four hair loss events; the estimate is statistically unstable and likely inflated, and should be interpreted with extreme caution. VIFs were below 1.25 and McFadden’s pseudo-R^2^ = 0.176.

## Data Availability

The data supporting the findings of this study are available from the corresponding author upon reasonable request.

## Abbreviations

The following abbreviations are used in this manuscript:

AA	Alopecia Areata
COVID-19	Coronavirus Disease 2019
SALT	Severity of Alopecia Tool
aOR	Adjusted Odds Ratio
TE	Telogen Effluvium
